# Additive analgesic effect of dexmedetomidine and dezocine administered intrathecally in a mouse pain model

**DOI:** 10.18632/oncotarget.25304

**Published:** 2018-05-11

**Authors:** Ya-Qin Huang, Shao-Hui Guo, Renyu Liu, Sheng-Mei Zhu, Jian-Liang Sun, Yong-Xing Yao

**Affiliations:** ^1^ Department of Anesthesia, First Affiliated Hospital, Zhejiang University School of Medicine, Hangzhou, P. R. China; ^2^ Department of Anesthesia, Hangzhou Hospital Affiliated With Nanjing Medical University, Hangzhou First People's Hospital, Hangzhou, P. R. China; ^3^ Department of Anesthesia, Second Affiliated Hospital of Wenzhou Medical University, Wenzhou, P. R. China; ^4^ Department of Anesthesiology and Critical Care, Perelman School of Medicine at The University of Pennsylvania, Philadelphia, PA, USA

**Keywords:** dexmedetomidine, dezocine, analgesia, acute nociception

## Abstract

**Background:**

It is known that dexmedetomidine can reduce opioid requirements and that there is a synergistic effect when dexmedetomidine and morphine (a full mu opioid receptor agonist) are administered together. However, it was unclear whether a synergistic or additive effect would be observed when dexmedetomidine was co-administered with a partial mu opioid receptor agonist. The present study was designed to elucidate such effects by intrathecally co-administering dexmedetomidine and dezocine, a partial mu receptor agonist, in a mouse pain model.

**Methods:**

C57 mice (N = 165) were randomly divided into 19 groups. The tail flick test was adopted to measure the antinociceptive effects of the tested agents. The mice were divided into saline and drug groups to investigate the dose-dependent analgesic effects. Each drug was administered at fixed doses alone and in combination with one of three doses of a second drug.

**Results:**

Dezocine (0.3125 - 1.25 μg) and dexmedetomidine (0.04 - 1 μg) both enhanced the tail withdrawal latency in dose-dependent fashions. Dexmedetomidine (0.04 - 1 μg) enhanced the analgesic effect of dezocine. Dezocine (0.3125 - 1.25 μg) enhanced the analgesic effect of dexmedetomidine. Compared with the individual drug effects, the combined effects of dezocine (0.625 μg) and dexmedetomidine (0.04 μg) were more potent 15 - 60 min after injection, but they remained similar to the sum of the effects of the two individual drugs.

**Conclusions:**

Dexmedetomidine and dezocine produce an additive analgesic effect on acute nociception when administered simultaneously.

## INTRODUCTION

Dexmedetomidine is a potent and highly selective agonist of α_2_-adrenergic receptors with analgesic properties against acute inflammatory pain [1], postoperative pain [2, 3], and even neuropathic pain that is unresponsive to opioid analgesics [4]. It has also been widely used in clinical settings as an adjuvant for conscious sedation [5–7]. Previous reports have revealed that dexmedetomidine could reduce opioid requirements and that there is a synergistic effect when dexmedetomidine and morphine (a full mu opioid receptor agonist) are administered together [8, 9]. However, it is unclear whether a synergistic or additive effect can be observed when dexmedetomidine is co-administered with a partial mu receptor agonist. Dezocine, a partial mu opioid receptor agonist and kappa opioid receptor antagonist, produces potent analgesic effects on acute and chronic pain [10–14]. Our recent reports and other studies have suggested that dezocine is at least as effective as morphine and can be used to control moderate to severe pain, such as postoperative pain [15, 16]. Since it is a partial mu opioid receptor agonist with potential ceiling effects on its responsive receptor, it could be used as a tool to investigate the potential additive or synergistic effects on pain with other medication(s).

In the present study, we investigated the effect of the intrathecal administration of dezocine and dexmedetomidine, two drugs widely used in the perioperative environment, on acute pain conditions *via* tail flick assays in mice to demonstrate that dezocine and dexmedetomidine have additive or synergistic analgesic effect on nociceptive stimulation.

## RESULTS

### Intrathecal dezocine or dexmedetomidine produce dose-dependent antinociception

**Figure 1:**
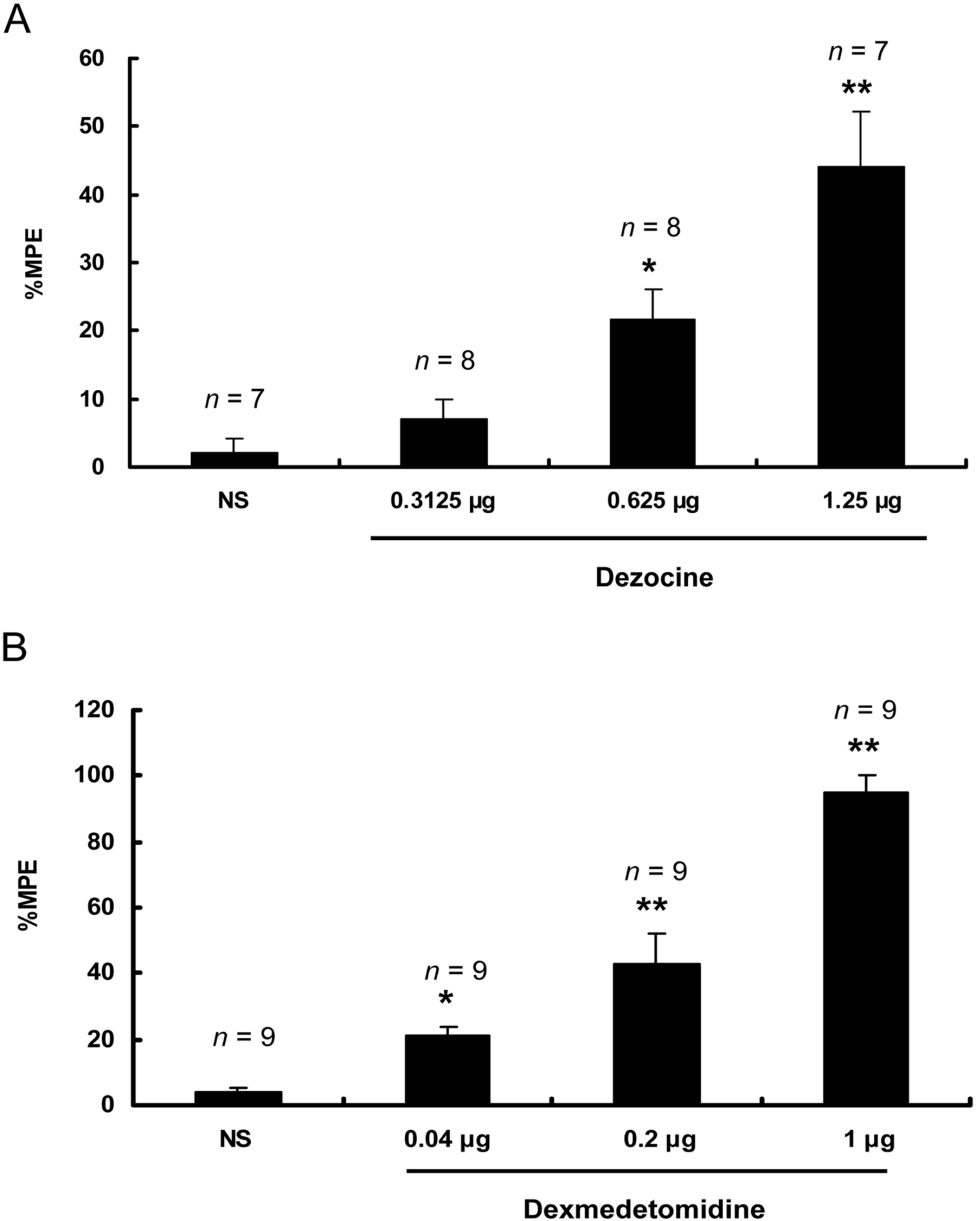
Intrathecal dezocine or dexmedetomidine produced dose-dependent antinociception in the tail flick test Within the 0.3125 - 1.25 μg dose range, 15 min after intrathecal injection, dezocine increased the tail withdrawal latency in a dose-dependent fashion **(A)**. Within the 0.04 - 1 μg dose range, dexmedetomidine increased the tail withdrawal latency in a dose-dependent manner **(B)**. ^*^P < 0.05, ^**^P < 0.01 *vs* the NS group. Error bars represent the SEM. NS = Normal saline. MPE = Maximum possible effect.

Compared with the saline control, dezocine or dexmedetomidine increased the tail withdrawal latency in a dose-dependent fashion, as measured at 15 min after intrathecal injection, within the dose range of 0.3125 - 1.25 μg of dezocine (0 μg, 2.04 ± 2.04 %; 0.3125 μg, 6.94 ± 2.92 %; 0.625 μg, 21.53 ± 4.48 %; 1.25 μg, 43.96 ± 8.15 %; ANOVA, *F* = 14.375, *P* = 0.000; *P* = 0.015 vs 0.625 μg, *P* = 0.009 vs 1.25 μg, *n* = 7-8, Figure 1A) and 0.04 - 1 μg of dexmedetomidine (0 μg, 3.7 ± 1.85 %; 0.04 μg, 21.03 ± 2.88 %; 0.2 μg, 42.92 ± 9.36 %; 1 μg, 95.06 ± 4.94 %; ANOVA *F* = 50.614, *P* = 0.000; *P* = 0.035 vs 0.04 μg, *P* = 0.000 vs 0.2 μg and 1 μg, *n* = 9-10, Figure 1B).

### Dose-dependent analgesic effect of dezocine potentiated by dexmedetomidine and vice versa

**Figure 2:**
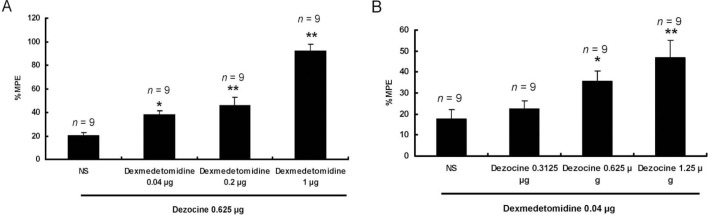
Dose-dependent analgesic effects of dezocine were potentiated by dexmedetomidine and vice versa At 15 min after intrathecal injection, dexmedetomidine increased the effect of dezocine in a dose-dependent fashion within a dose range of 0.04 - 1 μg **(A)**. At 15 min after intrathecal injection, dezocine increased the effect of dexmedetomidine in a dose-dependent fashion within a dose range of 0.3125 - 1.25 μg **(B)**. ^*^P < 0.05, ^**^P < 0.01 *vs* the NS group. Error bars represent SEM. NS = Normal saline. MPE = Maximum possible effect.

When a fixed dose of 0.625 μg dezocine was administered, dexmedetomidine increased the effect of dezocine in a dose-dependent fashion, within a dose range of 0.04 - 1 μg (0 μg, 20.26 ± 2.4 %; 0.04 μg, 38.03 ± 3.01 %; 0.2 μg, 45.92 ± 6.91 %; 1 μg, 92.28 ± 5.35 %; ANOVA, *F =* 41.336, *P* = 0.000; *P* = 0.013 *vs* 0.04 μg, *P* = 0.001 *vs* 0.2 μg, *P* = 0.000 *vs* 1 μg, *n* = 9, Figure 2A). When a fixed dose of dexmedetomidine was administered, dezocine increased the effect of dexmedetomidine in a dose-dependent fashion, within a dose range of 0.3125 - 1.25 μg (0 μg, 17.72 ± 4.33 %; 0.3125 μg, 22.47 ± 3.89 %; 0.625 μg, 35.58 ± 4.79 %; 1.25 μg, 46.69 ± 8.35 %; ANOVA, *F =* 5.433, *P* = 0.004; *P* = 0.032 *vs* 0.625 μg, *P* = 0.001 *vs* 1.25 μg, *n* = 9, Figure 2B).

### Additive effect of dezocine combined with dexmedetomidine in the tail flick assay

**Figure 3:**
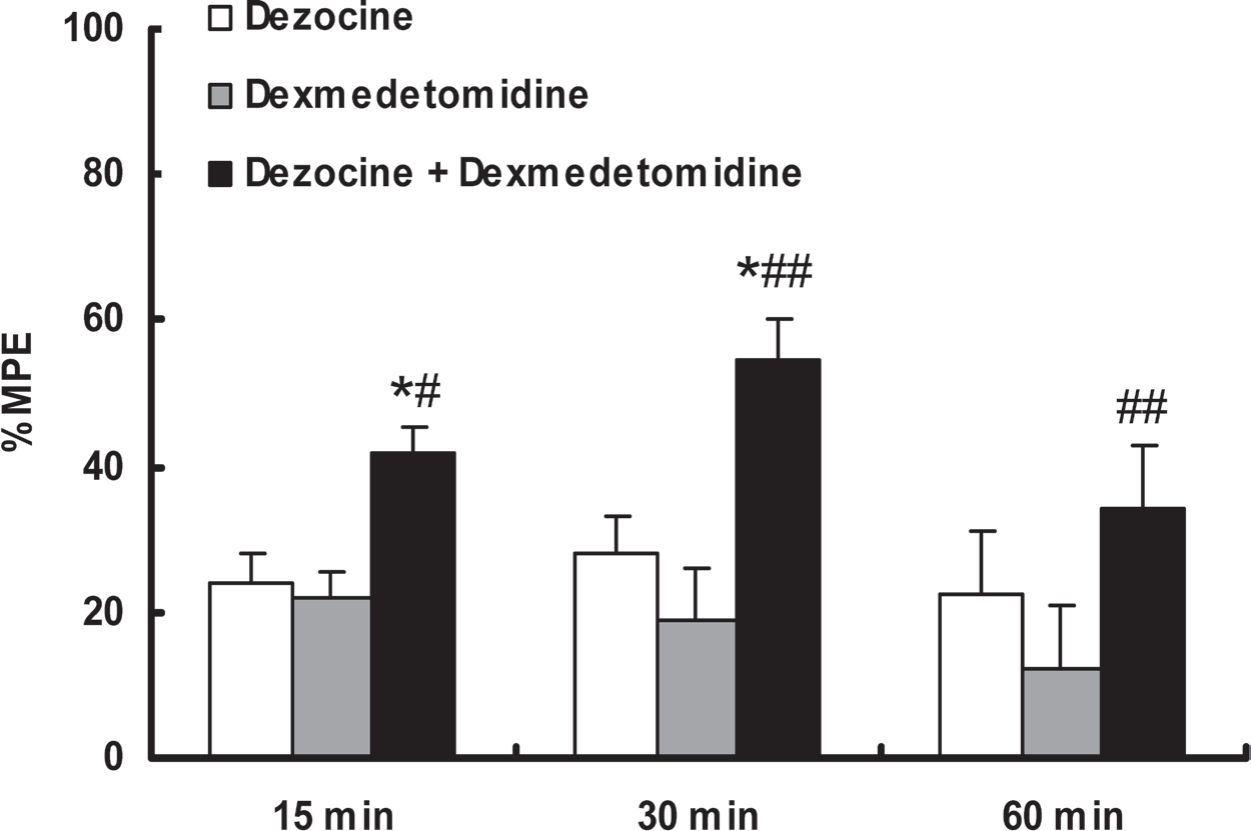
Additive effect of dezocine combined with dexmedetomidine in a tail flick assay At 30 minutes after injection, the combined effect of dezocine (0.625 μg) and dexmedetomidine (0.04 μg) was more potent than the effect of dezocine or dexmedetomidine alone. However, this effect remained similar to the sum of the two individual effects. ^*^P < 0.05 *vs* dezocine, ^#^*P* < 0.05, ^##^*P* < 0.01 *vs* dexmedetomidine, *n* = 9. Error bars represent the SEM. NS = Normal saline. MPE = Maximum possible effect.

The combined effect of dezocine and dexmedetomidine was more potent at 15 min (37.96 ± 4.46 %; *P* = 0.043 *vs* dezocine, 23.96 ± 4.11 %; *P* = 0.022 *vs* dexmedetomidine, 21.96 ± 5.24 %; *n* = 9, ANOVA, *F* = 3.550, *P* = 0.045), 30 min (47.53 ± 7.09 %; *P* = 0.034 *vs* dezocine, 28.17 ± 3.73 %; *P* = 0.0032 *vs* dexmedetomidine, 18.98 ± 6.81 %; *n* = 9, ANOVA, *F =* 5.762, *P* = 0.009), and 60 min (33.17 ± 4.76 %; *P* = 0.005 *vs* dexmedetomidine, 12.04 ± 5.66 %; *n* = 9, ANOVA, *F =* 4.907, *P* = 0.016) after administration. However, the effect remained similar to the sum of the two individual effects (37.96 ± 4.46 % *vs* 45.92 ± 7.17 %, *t* = 0.942, *P* = 0.360 at 15 min; 47.53 ± 7.09 % *vs* 47.15 ± 9.61 %, *t* = −0.032, *P* = 0.975 at 30 min; 33.18 ± 4.76 % *vs* 34.46 ± 7.58 %, *t* = 0.143, *P* = 0.888 at 60 min) (Figure 3).

## DISCUSSION

Postoperative pain is the most frequently encountered complaint in postoperative units, and overdoses of opioid analgesics increase the incidence of respiratory depression, hypoxia, and even cardiac arrest. Multi-model analgesic strategies not only reduce the opioid requirement but also reduce the side effects of each medication component.

Pharmacological intervention remains the mainstay for the control of acute pain. In this study, we not only demonstrated that intrathecally administered dezocine or dexmedetomidine alone dose-dependently increased the pain threshold of nociceptive heat stimulation but also found that when added to a fixed dose of dezocine, dexmedetomidine dose-dependently potentiated the analgesic effect of dezocine. When added to a fixed dose of dexmedetomidine, dezocine potentiated the analgesic effect of dexmedetomidine in a dose-dependent fashion. Moreover, we found that the combined use of dezocine with dexmedetomidine produced an effect that was greater than either of their individual effects alone and that was similar to, but not greater than, the sum of their individual effects. Therefore, we deduce that the effect produced by these two drugs, at least at the present doses, is additive rather than synergistic or antagonistic, although we cannot rule out the possibility of other interaction patterns at very low or very high doses.

Once known as a mu opioid receptor antagonist and kappa opioid receptor agonist, dezocine has recently been recognized as a weak mu opioid receptor agonist and kappa opioid receptor antagonist, with an inhibitory effect on the re-uptake of noradrenalin and serotonin [10, 11, 17, 18]. Several reports have suggested that dezocine possesses a potent analgesic effect, and thus, it has been used in perioperative pain management [19, 20]. It has been demonstrated that dezocine's analgesic effect at a dose of 10 mg is the same as that of either 50 mg of meperidine or 10 mg of morphine [14]. However, there have been no reports regarding the effect of the interaction between dezocine and the α_2_-adrenergic receptor agonist dexmedetomidine on acute nociception when co-administered intrathecally. Since the use of opioids combined with other medication(s) remains a common practice to control surgical pain, an understanding of the interactive effects of the drugs could be beneficial to patients by enhancing analgesia and reducing side effects. In this study, we discovered that intrathecal co-administration of dexmedetomidine and dezocine produced additive analgesic effects, indicating that each drug acts through different receptors in the spinal cord, which is consistent with the known pharmacological effects of both medications.

Dexmedetomidine and dezocine belong two different categories of medications with completely different mechanisms involving different receptors related to pain. Using an additive/synergistic assay, we demonstrated that dezocine combined with dexmedetomidine produced an additive, but not an antagonistic or synergistic, effect on the tail flick test in mice. Thus, it is predicted that dexmedetomidine could reduce the requirements of opioids for analgesic purposes. While both medications produce analgesic effects through receptors in the brain, they also have targets in the spinal cord. This study clearly indicates that they can generate additive effects at the spinal cord level. Although both opioid receptors and alpha-adrenergic receptors belong to the G protein-coupled receptor family, they play different roles in pain perception. Thus, an additive analgesic effect is somewhat more likely than other interaction patterns. While the analgesic effects of the two drugs are additive, the drugs are not interchangeable. Studies have indicated that there is a synergistic potentiation of antinociceptive effects mediated by morphine and clonidine (an agent similar to dexmedetomidine) [21, 22, 23], although there have been reports showing that adding dexmedetomidine to spinal anesthesia could result in longer motor and sensory blocks than the addition of narcotics [24]. The major difference between morphine and dezocine as pain medication is that morphine is a pan-agonist that activates all the opioid receptors, including kappa opioid receptors, while dezocine is a partial mu receptor agonist and kappa opioid receptor antagonist. It is unclear whether dexmedetomidine interacts with opioid receptors in the spinal cord. If dexmedetomidine interacts with opioid receptors, especially kappa opioid receptors, as a full agonist, it would be potential mechanism to explain why morphine and dexmedetomidine have synergistic effects. Molecular target studies need to be performed to investigate that possibility. In clinical practice, additive effects may be more desirable than synergistic effects, since they are more predictable, making it easier to avoid undesirable side effects. However, the present results were obtained in animal models, and clinical studies are necessary to further elucidate the interactions and the potential side effects, such as over-sedation or respiratory depression, of this combination of drugs.

## MATERIALS AND METHODS

Animal use and care, as well as the experimental protocols, were reviewed and approved by the Ethics Committee of the First Affiliated Hospital, Zhejiang University School of Medicine. These studies were also consistent with the ethical guidelines for the investigation of experimental pain in animals [25]. All possible efforts were made to minimize the number of animals used and their suffering.

### Animals

Male adult C57 mice (N = 165; body weight ~25 g) were obtained from the Animal Center of the Chinese Academy of Sciences (Shanghai, P. R. China). The animals were housed with 10 mice per cage with water and food available *ad libitum* and were maintained under a 12-h light/12-h dark cycle with the lights turned on at 08:00 AM.

### Tail flick test

The tail withdrawal latency was assessed as previously reported [26]. Briefly, the mice were gently restrained along the edge of a table, and their tails were immersed in water, which was maintained at 52°C using hot water baths. The latency to tail withdrawal was measured using a hand-held stopwatch. One hour after the baseline was determined, medications (5 μl in total volume) were intrathecally administered via direct lumbar puncture, according to the procedure described by Hylden and Wilcox in 1980 [27]. Briefly, a modified 25 μl micro-syringe was inserted between the L5 and L6 vertebrae of conscious mice. A sudden flick of the tail indicated the success of the puncture into the subarachnoid space. The drug solution or vehicle was injected over a period of 30 s. The tail withdrawal latency was re-determined at 15, 30 or 60 min after drug administration. To avoid tissue damage, a maximum score (100 %) was assigned to the animals that did not respond within 10 s. Antinociception was calculated by the following formula: % maximum possible effect (MPE) = 100 × (test latency - baseline latency) / (10 - baseline latency) [28].

### Medications

Dezocine was obtained from the Yangtze River Pharmaceutical Group Co., Ltd., Jiangsu, P. R. China. Dexmedetomidine was obtained from the Jiangsu Hengrui Medicine Co., Ltd., Jiangsu, P. R. China.

The animals were randomly allocated to each group. The sample size was estimated according to previous reports [28, 29]. Both dezocine and dexmedetomidine were administered intrathecally. To determine the dose-dependent effects on antinociception, the mice were divided into saline control, dezocine (0.3125, 0.625, or 1.25 μg), or dexmedetomidine (0.04, 0.2, or 1 μg) groups. To investigate the interaction between dezocine and dexmedetomidine, dezocine was administered at a fixed dose of 0.625 μg and combined with various doses of dexmedetomidine (0.04, 0.2, or 1 μg) in one set of experiments. Dexmedetomidine was administered at a fixed dose of 0.04 μg and combined with various doses of dezocine (0.3125, 0.625, or 1.25 μg) in another set of experiments. The additive/synergistic effect was deduced via comparison of the combined effect with the sum of the two individual effects, according to previous reports [16, 29–31].

### Statistical analysis

The data are expressed as the mean ± standard error of the mean (SEM) and were analyzed with one-way analysis of variance (ANOVA), followed by the least significant difference test for multiple comparisons (LSD). To test additivity in the one-dose experiments, the mean values and standard variations of the combined single-drug groups were compared with the values of the respective combination group using independent *t*-tests. A *P*-value < 0.05 was considered statistically significant.

## CONCLUSIONS

In conclusion, dezocine and dexmedetomidine produce dose-dependent antinociceptive effects in mice. When simultaneously administered intrathecally, dezocine and dexmedetomidine produce an additive effect on acute nociception. These results suggest that dexmedetomidine can be used to enhance the analgesic effects of dezocine and/or to reduce the requirement for dezocine for acute pain management in the perioperative period.
